# An Individual-Oriented Model on the Emergence of Support in Fights, Its Reciprocation and Exchange

**DOI:** 10.1371/journal.pone.0037271

**Published:** 2012-05-30

**Authors:** Charlotte K. Hemelrijk, Ivan Puga-Gonzalez

**Affiliations:** Behavioural Ecology and Self-Organization, Centre for Ecological and Evolutionary Studies, University of Groningen, Groningen, The Netherlands; Hungarian Academy of Sciences, Hungary

## Abstract

Complex social behaviour of primates has usually been attributed to the operation of complex cognition. Recently, models have shown that constraints imposed by the socio-spatial structuring of individuals in a group may result in an unexpectedly high number of patterns of complex social behaviour, resembling the dominance styles of egalitarian and despotic species of macaques and the differences between them. This includes affiliative patterns, such as reciprocation of grooming, grooming up the hierarchy, and reconciliation. In the present study, we show that the distribution of support in fights, which is the social behaviour that is potentially most sophisticated in terms of cognitive processes, may emerge in the same way. The model represents the spatial grouping of individuals and their social behaviour, such as their avoidance of risks during attacks, the self-reinforcing effects of winning and losing their fights, their tendency to join in fights of others that are close by (social facilitation), their tendency to groom when they are anxious, the reduction of their anxiety by grooming, and the increase of anxiety when involved in aggression. Further, we represent the difference in intensity of aggression apparent in egalitarian and despotic macaques. The model reproduces many aspects of support in fights, such as its different types, namely, conservative, bridging and revolutionary, patterns of choice of coalition partners attributed to triadic awareness, those of reciprocation of support and ‘spiteful acts’ and of exchange between support and grooming. This work is important because it suggests that behaviour that seems to result from sophisticated cognition may be a side-effect of spatial structure and dominance interactions and it shows that partial correlations fail to completely omit these effects of spatial structure. Further, the model is falsifiable, since it results in many patterns that can easily be tested in real primates by means of existing data.

## Introduction

When observing complex behaviour of animals, we automatically attribute it to sophisticated cognitive mechanisms. This is usually accepted when observing intelligent animals, such as primates and humans [1], but not in the case of social insects, when we study, for instance, the complex organization of their large colonies [2] or the highly sophisticated architecture of their nests, such as termite hills [3]. The cognitive complexity of insects is known to be limited and, therefore, complexity of traits is thought to arise by self-organization [4], [5]. However, more recently, complex traits in taxa with great cognitive sophistication have also increasingly been considered to be due to self-organization based on cognitively simple behavioural rules [6]–[8]. This even includes patterns of behaviour in humans, such as the segregation of races [9] and the complexity of financial markets [10]. This means that it is difficult to tell what part of the complex spontaneous behaviour of highly intelligent animals, such as primates, is due to cognitive sophistication and what part is due self-organization [11].

In the present paper, we demonstrate in a computer model that among agents with minimal cognition, patterns of coalitions emerge from grouping, dominance interactions, and grooming through self-organization. These cognitively simple agents appear to form coalitions, show patterns usually thought to indicate triadic awareness in the choice of coalition partners, and reciprocate support in fights and exchange it for grooming.

More than any other behaviour, coalition formation has been thought to reflect the cognitive sophistication of primates [12]. Recruitment of support is believed to involve awareness of the social relationships between other individuals in connection with the relations between the individual itself and these other individuals, so-called ‘triadic awareness’ [12]–[18]. Support in fights and grooming have been regarded as altruistic and according to the framework of reciprocal altruism, their receipt should be repaid in return [19] by cognitively keeping track of the number of acts given to, and received from each partner, so-called calculated reciprocity [20]–[22]. Calculated reciprocity was suggested to be most complicated in cases where individuals reciprocated not only their support but also their opposition towards others (called contra-support), showing so-called spiteful behaviour [20].

The necessary involvement of sophisticated cognitive abilities in reciprocation is a point of view that is not adhered to by all scientists. For example, Range and Noë [23] argue that in recruiting support, individuals may simply recruit others of higher rank than themselves and no triadic awareness is needed. Stevens and colleagues [24]–[26] contend that food sharing reflects tolerated theft [27] and that calculated reciprocity has so far not been shown [28]. Others suggest that coalition behaviour may involve simple rules of thumb [29], that its reciprocation and exchange may emerge as a side-effect from opportunistic attacks [30]–[32] or involve a kind of emotional book-keeping [33] and that chimpanzees are not able to show spiteful behaviour, but that they merely retaliate [34]. In agreement with this, animal taxa with supposedly lower cognitive abilities, such as hyenas, appear to show patterns of coalition behaviour and reciprocation similar to primates [35].

**Figure 1:**
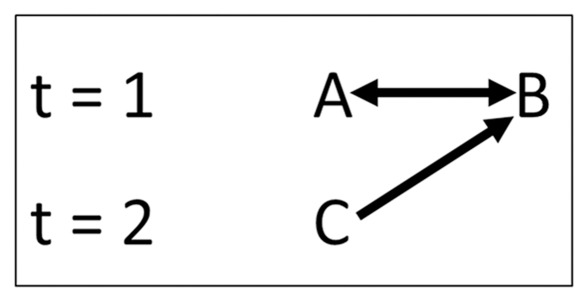
Coalitions in GrooFiWorld. At time 1, individuals *A* and *B* are fighting. At time 2, individual *C* attacks *B* and hereby supports *A* and opposes *B* (contra-support). Individual *C* is the supporter and individual *B* is the target (see video S1).

In our study, we avoid this debate on what intelligence underlies complex social behaviour in primates. Instead, our study is part of a broader research program, in studies of humans and animals, also called the ‘low-intelligence approach’ [10] or that of ‘minimal cognition’ [7], in which ‘null-models’ are developed for complex patterns of behaviour. We use an earlier computer model [36] to investigate whether patterns of coalition, such as reciprocation of support and the exchange between support and grooming, may result through self-organization due to aversion of risks of attack, anxiety-reducing effects of grooming and socio-spatial structuring. We give individual agents ‘minimal cognition’: individuals aggregate and when they are too close to others, they are more likely to attack them if they are under the impression that they will win [37], [38]. Winning and losing has self-reinforcing effects [39]–[42]. However, when individuals fear defeat, they will tend to groom the other individual, particularly when they are anxious [36]. Coalitions may emerge in the model as a consequence of ‘social facilitation’, i.e., an individual C close to a fight is activated sooner than another individual that is further away. Such spatial proximity (e.g., C being close to the two combatants, A and B, Figure 1) may incidentally result in support in the fight when an individual (C) attacks one of two combatants (e.g., B), because this is counted as an act of support (for A) and opposition (to B)(also called contra-support), as is done when recording behaviour of real primates [20], [30], [31], [43]–[50]. In the present paper, we will refer to contra-support by the word ‘opposition’.

In our present study, we first derive predictions for our model by means of a survey of empirical patterns of coalition (Table 1). Primate species have been shown to differ in dominance style or type of society, often classified as egalitarian and despotic, with different gradations [51], [52]. Since dominance style has been shown to influence patterns of both aggression and grooming [51], [53], [54], we also study the relationship between dominance style and coalitions in the model. In primates, the most detailed comparison between despotic and egalitarian species has been made in the genus of macaques. Here, despotic species differ from egalitarian ones in several traits: they have a steeper hierarchy, lower frequency of aggression, more asymmetrical aggression, greater dominance of females over males [42], a lower conciliatory tendency [51], [53], and more grooming up the hierarchy and of others of similar rank [36].

**Table 1:** Coalition patterns among adult females in Old-World primates.

Species	Dominance style	# Subjects (Group size)	% Fem in group	Coalitions as % of fights	% of coalition types	Reciprocity of support	Exchange of support:		Reciprocity of opposition	Sources
							Grooming for support	Support for grooming		
1) *M. sylvanus*	1E	51(250)	--	--	--	--	--	--	--	[118]
2) *M. radiata*	1E	10(72–80)	80%	--	R:9%	2NO	NO	--	--	[46]
3) *M. assamensis*	1D	10(64)	52%	15%	--	--	3YES	--	--	[48], [119]
4) *M. fascicularis*	1D	21(30–40)	--	--	--	--	YES	--	--	[120]
5) *M. fuscata*	1D	18(37)	--	6%	C:70% B:26% R:4%	--	--	--	--	[121]
6) *M. fuscata*	1D	Nina group: 8(25)	--	--	--	--	--	YES	--	[122]
7) *M. fuscata*	1D	Kw group: 20(55)	--	--	--	--	--	YES	--	[122]
8) *M. fuscata*	1D	23(57)	--	6%	--	YES	YES	YES	--	[50]
9) *M. mulatta*	1D	12(?)	--	--	--	--	YES	--	--	[123]
10) *M. mulatta*	1D	34(172)	53%	--	--	--	--	--	--	[43]
11) *C. aethiops*	NA	Group A: 8(29)	53%	--	--	--	YES	--	--	[124]
12) *C. aethiops*	NA	Group B: 7(17)	70%	--	--	--	NO	--	--	[124]
13) *C. aethiops*	NA	Group C: 8(29)	66%	--	--	--	YES	--	--	[124]
14) *P. cynocephalus*	NA	Linda: 11(NA)	--	4%	C:76%	2NO	2NO	--	--	[44]
15) *P. cynocephalus*	NA	Nyayo: 17(NA)	--	1%	C:76%	2NO	2NO	--	--	[44]
16) *P. cynocephalus*	NA	Omo: 7(NA)	--	3%	C:76%	2NO	2NO	--	--	[44]
17) *P, cynocephalus*	NA	Viola: 10(NA)	--	3%	C:76%	2NO	2YES	--	--	[44]
18) *P. cynocephalus*	NA	Weaver: 14(NA)	--	2%	C:76%	2YES	2NO	--	--	[44]
19) *P. cynocephalus*	NA	8(24)	80%	7%	--	YES	--	--	--	[125]
20) *P. hamadryas ursinus*	NA	13(70)	75%	4.4/10.5^4^	--	--	--	--	--	[126]
21) *T. gelada*	NA	3–6(NA)	--	--	--	YES	--	--	--	[127]
22) *P. troglodytes*	NA	16(20–30)	64%	--	--	YES	YES	YES	NO	[30]

Coalition types: C:Conservative, B:Bridging, R:Revolutionary [55]. NA: not available. --: not tested.

1 [51].

2 Only partial Tau-Kr value reported.

3 Calculated here using published data.

4 Physical support/physical and vocal support.

We have shown in earlier studies that this modelling approach produces both the patterns of aggression, grooming and conciliatory behaviour exhibited by many primate species and the differences between egalitarian and despotic species of macaques [7], [36], [38], [42], while in our present study, we demonstrate that these findings still hold for a larger group size (of 30 instead of 12 individuals) [36]. Furthermore, we show that such an approach also leads to surprisingly good predictions regarding new patterns: different types of coalitions, i.e., conservative, bridging or revolutionary coalitions [55], indications of triadic awareness in the choice of coalition partners, reciprocation of support and opposition and exchange between support and grooming. We deliver predictions to verify our findings.

## Methods

### Survey of empirical data

To compare the results of the model to empirical data, we surveyed the literature on coalitions in primates (Table 1). We confine ourselves to adults and to Old World primates, because New World primates differ in their patterns of social interactions, e.g., in the percentage of the time spent grooming and in their patterns of grooming [56]–[59]. We only included studies of coalition patterns among individuals of the same sex. We surveyed 26 studies, comprising 31 groups of 13 different species (Table 1).

### The model

In our model, called GroofiWorld [36], we represent the essential traits of primate societies: individuals group and they compete in the group for unspecified reasons (Fig. S1). In this competition, the effects of winning and losing are self-reinforcing [39], [40], [60], [61] and individuals try to avoid the risk of losing a fight [62]. When risks are high, individuals will tend to avoid the risk of losing a fight by grooming the other individual instead. Thus, individuals first consider fighting and then grooming. This order is based on the observation by Kummer that unfamiliar individuals will first fight with each other and then groom [63]. Individuals in the model become more anxious after a fight, as indicated in reality by the increase in frequency of scratching and heart rate in both opponents [64]–[71]. In addition, in the model, their anxiety may subsequently be reduced by the receipt of affiliative behaviour and, to a lesser degree by active grooming, as indicated by the reduced heart rate and the drop in the rate of self-directed behaviour in many species [68], [69], [71]–[73]. Furthermore, our model is informed by empirical studies on grooming and opiate administration which indicate that not being groomed for some length of time reduces the concentration of endorphins and increases the motivation to be groomed, and that grooming increases the level of endorphins in the brain and reduces the motivation to groom [74]–[79]. Individuals are activated in random order, but if an individual is close to a fight, i.e., within a certain radius (see Table 2 for radius of social facilitation), then it may be activated earlier, i.e., through social facilitation (Table 2). We refer to Text S1 and our earlier paper for more details of the model [36]. Below, we describe the way in which coalitions were recorded in the model, how parameters were set, and analyses and experiments were carried out.

**Table 2:** Default parameter values in ‘GrooFiWorld’.

Parameter	Description	Females	Males
**General Parameters**			
GroupSize	Total number of individuals	30	
Sex ratio (at high aggression intensity)	Number of	24	6
Sex ratio (at low aggression intensity)	Number of	21	9
InitRadius	Predefined space at start of simulation	1.7*# Inds	1.7*# Inds
Radius of social facilitation	Radius starting from centre point between two opponents	10	10
**Grouping Parameters**			
PersSpace	Close encounter distance	8	8
NearView	Medium distance	24	24
MaxView	Maximal viewing distance	50	50
SearchAngle	Turning angle to find others	90°	90°
VisionAngle	Angle of field of view	90°	90°
**Dominance Parameters**			
InitDom	Initial Dom value	16	32
RiskAvers (high intensity)	Number of ‘mental battles’	∼2 (Eq. 1)	∼2 (Eq. 1)
RiskAvers (low intensity)	Number of ‘mental battles’	∼1 (Eq. 1)	∼1 (Eq. 1)
StepDom (high intensity)	Scaling factor for aggression intensity	0.8	1
StepDom (low intensity)	Scaling factor for aggression intensity	0.08	0.1
FleeingDistance	After losing a fight	2	2
ChaseDistance	After winning a fight	1	1
**Grooming Parameters**			
InitAnx	Initial anxiety value	0.5	0.5
AnxInc	Increase in anxiety after every activation	1%	1%
AnxDcrGree	Decrease of anxiety of groomee	0.15	0.15
AnxDcrGrmr	Decrease of anxiety of groomer	0.1	0.1
AnxIncFight	Increase of anxiety after fighting	0.1	0.1

### Coalitions

If two individuals attack the same target in two subsequent activations, this is classified as an event of coalition and opposition (Fig. 1; video S1).

### Parameters

Where possible, we kept the parameters of the model (Table 2) the same as in our previous studies [36], [80]. However, in order to also study interaction patterns among males in the future (Puga-Gonzalez et al, in prep), and given that the number of males in primate groups is lower than that of females, we used a larger group size to reach the minimal sample size of four that is required for the statistical analysis of males. Empirical studies show that the percentage of males in groups is approximately 30% in egalitarian primates and approximately 20% in despotic primates [81]–[83]. Therefore, our group size of 30 individuals included 21 females and 9 males at low intensity and 24 females and 6 males at high intensity. As a consequence of increasing the group size to 30 individuals, one empirical pattern was no longer met: the percentage of time spent fighting among females was no longer lower at high intensity of aggression when compared to low intensity of aggression [51], [84]. We solved this problem by increasing the risk-aversion of an individual, *RiskAvers*, when its opponent's intensity of aggression was higher (Equation 1). Consequently, the percentage of time spent fighting was lower at high intensity of aggression than at low intensity of aggression, in accordance with empirical data. Here, the average number of ‘mental’ battles at high intensity of aggression was ∼2 and at low intensity, ∼1.
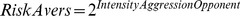
(1)


### Experimental set-up

We performed four experiments to understand what caused the patterns of coalition in the model. First, we switched off ‘social facilitation’ (i.e., the shortening of the waiting-time of those individuals close to a dominance interaction). Thus, when social facilitation is off, individuals close to a fight are as likely to be activated next as any other individual. Second, we disabled rank differences among individuals by randomly shuffling Dom values among all individuals after every activation. We used fixed Dom values (thus switching off the self-reinforcing effects). We took these Dom values for the corresponding intensity of aggression from the middle of the interval in which the Dom values were considered to have stabilized, thus, from between periods 200 and 260 (i.e., period 230) [85]. Third, we investigated the role of non-random spatial structure by making individuals interact with randomly chosen partners. Fourth, we investigated the role of the combination of spatial structure and rank by disabling them simultaneously. See Table S1 for further experimental manipulations of the behavioural rules (taking out the effect of anxiety on grooming, adjusting the probability of attacking other individuals to 28% at high intensity and 42% at low intensity (percentages are adjusted such that the same percentage of fights results as in the full model), independent of the risks involved, and reversing the order of behavioural rules concerning aggression and grooming and randomizing the order).

### Data collection and analysis

Every run consisted of 260 periods and each period consisted of 600 activations (i.e., GroupSize times 20). Data were collected from period 200 to 260 to exclude any bias caused by transient values. Data consisted of spatial position and direction of each individual and, for coalitions, fights and grooming behaviour of: 1) the actor and receiver and of the winner and loser and 2) the Dom values and degree of anxiety. For each condition (the complete model, and the models without one or more assumptions), 10 independent replicas were run for each of the two aggression intensities (high and low). The results are shown as the average value of the statistic over 10 runs for each condition. Their combined probability is based on the improved Bonferroni procedure [86]. We used non-parametric statistics and two-tailed probabilities. We only used one-tailed probabilities if patterns were predicted by empirical studies.

The percentage of time individuals spend fighting (or grooming) was calculated by dividing the total number of fights (or grooming bouts) by the total number of activations. Similar to empirical studies, the percentage of coalitions was calculated as the total number of coalitions divided by the total number of fights [44], [50].

The rank of group members was calculated as the average Dom value for each individual per run over periods 200–260. We used an average measure because we correlated it with an average measure of aggressive and affiliative acts, i.e., data were summed over the whole interval of period 200–260.

The hierarchical differentiation among individuals was measured by the coefficient of variation of Dom values for the average rank of each individual over period 200–260 and this was averaged over 10 runs. Higher values indicate greater rank distances between individuals [38]. Hierarchical differentiation is also reflected in the empirical behavioural measure of the degree of unidirectionality of aggression, which we present as well [51], [87].

The degree to which dominant individuals of a certain sex occupy the centre of the group was measured by a correlation between an individual's average Dom value and the average spatial direction of others around it. The centrality of each individual is calculated by means of circular statistics by drawing a unit circle around ‘ego’ and projecting the direction of other group members as points on the circumference of this circle [88]. The connection of these points with ego's location results in vectors. The length of the average vector represents the degree to which group members form a cluster relative to ego. Thus, longer mean vectors indicate a more peripheral, and hence, less central location of ego. The centrality of dominants is therefore represented by a negative correlation between rank and the length of the average vector (indicating the average direction of other individuals).

Correlations between the distribution of grooming, aggression, support and opposition among individuals, and between social interactions and rank and proximity were computed by means of the Tau-Kr correlation, as described by Hemelrijk [87], [89]. Matrices of support (and opposition) were corrected for opportunity (number of fights) to support (or oppose) each partner. Matrices of proximity were constructed using the average distance between individuals. All matrices were based on data collected over the supposedly stable periods from 200 to 260. The level of significance was calculated using 2000 permutations [87], [89]. We tested for reciprocity and exchange of attack, grooming, support and opposition by correlating an actor and receiver matrix with the Tau-Kr correlation [89]. To compare our results to those for real primates, we investigated the possibility that correlations were a side-effect of a correlation with a third variable by partialling it out using partial Tau-Kr correlations [87]. The third variables concerned rank and proximity.

Whether social behaviour (i.e., grooming, aggression, support and opposition) was directed up the hierarchy or towards partners of similar rank was computed, respectively, using the Tau-Kr correlation between the matrix of social behaviour and the matrix of the rank of partners (with the average Dom values of partners in the rows) and the matrix of partners of similar rank (filled with zeros apart from the partners closest and second closest in rank, which are indicated as 1's). Note that higher-ranking individuals have higher Dom values. Thus, a significant positive correlation corresponds to social behaviour being directed up the hierarchy and towards individuals of similar rank, respectively.

Because of the high number of correlations, significant results may arise by chance. We corrected for this in two ways. We used the Bonferroni correction and discarded the 5% of the lowest significances (Type I error) per table of results.

## Results

### Empirical patterns

In our survey of the empirical literature on coalitions in primates, we focus on females because they have been studied more often than males (in 22 studies versus 14 studies on males).These results serve as predictions for our models. Our survey shows that, on average, adult females form coalitions in 5% of their fights (based on 10 studies, Table 1), that these coalitions are most often conservative (all-down), less often bridging and least often revolutionary (all-up, 16–18 in Table 3), and that they reveal patterns that have been attributed to triadic awareness in the choice of coalition partners (19–21 in Table 3). This is inferred when individuals solicit support from others that are higher in rank than either they, themselves, or their opponent, even if the solicitor ranks below the opponent [13], [17], and when individuals (independent of their rank relative to the opponent) solicit support from others with a better relationship with them than with their opponent [13], [17]. Further, adult females reciprocate support at a group level in 50% of the studies (5/10), or 100% when excluding the studies based on partial correlations [44], [46], they exchange support for receipt of grooming in 100% (4/4) of the studies and they groom for receipt of support in 57% (8/14) (or 78% when excluding partial correlations: [44]) of the studies (Table 1). Reciprocation of opposition was tested among adult females in a single study only, namely in chimpanzee females, and appeared to be absent [30]. Whether results differ between dominance style, i.e., egalitarian and despotic, cannot be tested due to the small sample size.

**Table 3:** Dominance, affiliation and coalition patterns among females: empirical data and GrooFiWorld.

	Empirical studies on macaques		GrooFiWorld	
Intensity of Aggression	Despotic	Egalitarian	High	Low
**Dominance Style**				
1) Gradient of the hierarchy (CV)^1^	NA	NA	0.72	0.36
Gradient of the hierarchy High > Low	NA		U = 100***	
2) Unidirectionality of Aggression (TauKr)	2True	2NS	−0.13**	0.51***
Unidirectionality of aggression High > Low	2True		U = 100**	
3) Time spent fighting (%)	2NA	2NA	13%	17%
Fighting % High<Low	2NA		U = 100***	
4) Relative female dominance	30.23	30.00	0.22	0.00
Relative female dominance High > Low	3True		U = 100***	
5) Average distance among all group members	2High	2Low	29	25
Average distance High<Low	2NA		U = 97***	
6) Centrality of Dominants (Tau)	2True	2NA	−0.40**	−0.10
Centrality High > Low	2NA		U = 100***	
**Affiliative patterns**				
7) Time spent grooming (%)	28–15	2NA	17	20
8) Conciliatory Tendency	27–18	220–50	21	31
Conciliatory tendency High<Low	2U = 66*		U = 100***	
9) Grooming Reciprocation (TauKr)	2True	2True	0.39***	0.54***
Grooming Reciprocation High<Low	2NA		U = 94***	
10) Grooming up the hierarchy (TauKr)	2True	2NS	0.34***	0.04
Grooming up the hierarchy High > Low	2True		U = 100***	
11) Grooming partners of similar rank (TauKr)	2True	2NS	0.13**	−0.01
Grooming partner of similar rank High > Low	2True		U = 100***	
12) Reconciliation with valuable partners (TauKr)	2True	2True	0.37***	0.11**
Reconciliation valuable partners High > Low	2NA		U = 78*	
**Coalition patterns**				
Intensity of Aggression	Despotic and Egalitarian combined^4^		High	Low
13) % of fights involving coalitions	55%/9%		10%	7%
14) % of triadic coalitions (3 individuals)	675%		96%	98%
15) % of tetradic coalitions (4 individuals)	625%		4%	2%
**Coalition types against adults**				
16) Conservative coalitions %	770%		71%	29%
17) Bridging coalitions %	726%		21%	27%
18) Revolutionary coalitions %	74%		8%	44%
Jonckheere-Terpstra test (C>B>R)			JT = 0***	JT = 205 NS
**Patterns related to triadic awareness**				
19) Recipient<Target<Supporter^8^	884%		+(67%)*** 15	−(24%)*** 15
20) Support given to ‘friend’^9^	967%		+(70%)*** 15	+(54%)* 15
21) Support given to ‘friend’^10^	10NA		+(69%)*** 15	+(53)%* 15
**TauKr correlations**				
22) Reciprocation of support (TauKr)	11True		0.38***	0.27***
23) Grooming for Support Received (TauKr)	12True		0.36***	0.29***
24) Support for Grooming Received (TauKr)	13True		0.29***	0.36***
25) Reciprocation of opposition (TauKr)	14NS	NA	−0.11**	0.29***

Coalition patterns: empirical results of egalitarian and despotic species are lumped except for the frequency of coalition types which are reported in a single study [121]. Results represent the average over 10 runs. P-value based on the Bonferroni correction:

* = p<0.05;

** = p<0.01,

*** = p<0.001.

1 Among all individuals.

2 See our previously analyzed empirical data in: [36];

3 [42].

4 These species include more than macaques, also baboons and chimpanzees.

5 Excluding vocal coalitions/including vocal coalitions.

6 [90].

7 [121].

8 [17]: This study concerns males and females combined;

9 [13];

10 Omitting support from the relationship quality index [13];

11 13,26,28,29 in Table 1;

12 2,6,8,9,13,14,18,19,20,29 in Table 1;

13 4,11,12,13,29 in Table 1.

14 [30].

15 Supporter higher ranking than target and recipient: + more frequent than chance; − less frequent than chance.

### Analysis of empirical coalition patterns in the model

With reference to the *percentage of fights* with coalitions, the model generates percentages of incidental support that resemble those in real primates if vocal coalitions are included (13 in Table 3), despite the absence of any rules for coalition-formation. Furthermore, the percentages are higher than those for empirical data from which vocal coalitions have been excluded (Mann-Whitney U: high intensity vs empirical data, n_1_ = 10, n_2_ = 9, U = 80, p<0.01; low intensity versus empirical data, n_1_ = 10, n_2_ = 9, U = 79, p<0.01). As is the case for empirical data, coalitions in the model appear to be triadic more often than polyadic, but the percentage of triadic coalitions (96%–98%, 14 in Table 3) is higher than for empirical data, at 75%, and that of polyadic coalitions is lower, at 2–4%, in the model than for empirical data, at 25% (15 in Table 3) [90].

At high intensity of aggression in the model, *coalition types* are most often conservative, sometimes bridging, and least often revolutionary (16–18 in Table 3), while at low intensity of aggression, coalitions are usually revolutionary and less often conservative or bridging (Mann-Whitney U test, n = 10; revolutionary vs conservative: U = 100 p<0.01; revolutionary vs bridging: U = 100, p<0.01; conservative vs bridging: U = 63, p>0.1).

In relation to *triadic awareness* of the choice of coalition partners (19 in Table 3), despite the absence of soliciting behaviour in our model, supporters appear mostly to be higher in rank than the receiver (i.e., the individual that could have solicited) and also than the target at high intensity of aggression, even if the receiver (‘solicitor’) ranks below its opponent. This resembles pooled empirical data for individuals of both sexes in studies on capuchin monkeys and Japanese macaques [13], [17]. Further, in agreement with empirical data, the relationship of the supporter – measured by the sociality index of Perry and co-authors [13]- is better with the receiver (‘solicitor’) than with the target in the model at both intensities (20, 21 in Table 3).

Females *reciprocate* support and *interchange* grooming for receipt of support and support for receipt of grooming at both intensities of aggression in the model (22–24 in Table 3). This resembles empirical data, but reciprocation of support and exchange of grooming for support are found at a higher frequency (100% vs 50% and 100% vs 57% respectively) in the model.

Supporting a certain individual in a triadic fight implies opposing the other individual. *Opposition* is reciprocated at low intensity of aggression (thus, individuals more often oppose those partners from whom they receive more opposition [87]) but not reciprocated at high intensity of aggression, resembling results for female chimpanzees [30], and it is even unidirectional (25 in Table 3). In addition to empirically-derived hypotheses, we also studied other correlations of opposition with grooming and support. At both intensities of aggression in the model, females oppose those individuals more frequently whom they support more often (11 in Table S2) and by whom they are groomed more often (10 in Table S2) and females receive opposition more often from those partners whom they groom and support more frequently (9, 12 in table S2). It thus appears that ‘services’ are exchanged for harmful acts.

There are several significant differences at a *high versus low intensity* of aggression: 1. The percentage of coalitions that is conservative is higher (high vs low intensity of aggression, Mann-Whitney U = 100, p<0.001) and the percentage that is revolutionary is lower (high vs low intensity of aggression, Mann-Whitney U = 100, p<0.001), 2. Individuals more frequently show ‘triadic awareness of choice of coalition partners at high than at low intensity, 3. The degree of reciprocity of support is greater (1 in Table S3), 4. The correlation for exchange of grooming for support is stronger and the correlation for support for grooming is weaker (20, 21 in Table 4; 2, 3 in Table S3), 5. Opposition is unidirectional at high intensity and bidirectional at low intensity of aggression (4 in table S3).

**Table 4:** Model-based hypotheses.

Model-based hypotheses for adult females:	Empirical data
**A) In general:**	
1) Revolutionary coalitions are more frequent the higher the percentage of males in the group	NA
2) In larger groups the conciliatory tendency is higher and the correlation for the valuable relationship hypothesis is stronger.	NA
3) The stronger the degree of social facilitation, the higher the frequency of support and the percentage of polyadic support	NA
4) The number of coalitions among females is higher the higher their percentage in the group	NA
Females:	
5) Groom those more often that they support more frequently	Pro: [30]
6) Receive grooming more frequently from those that they more often receive support from	NA
7) Receive aggression more often from those that they more frequently receive opposition from	NA
8) Aggress those more often that they oppose more frequently	NA
9) Groom those more often that they more frequently receive opposition from	Contra: [30]
10) Oppose those more often that they more frequently receive grooming from	NA
11) Oppose those more often that they more frequently support	NA
12) Support those more often that they more frequently receive opposition from	NA
**B) In egalitarian species:**	
13) Opposition is bidirectional	Contra: [20]
**C) In despotic species:**	
14) Females receive support more frequently from partners, the higher the rank of their partner	Pro: [30]
15) Opposition is unidirectional	Pro: [20]
16) Supporters are significantly more often higher ranking than the target of the coalition, even if the recipient of support ranks below the target	Pro: [17], [23]
**D) In despotic compared to egalitarian species** 1	
17) Coalitions are less often revolutionary	NA^1^
18) Females will more often solicit others that are higher in rank than both the solicitor and target.	NA
the correlation at a group level for:	
19) reciprocation of support is stronger	NA
20) the exchange of grooming for support is stronger	NA
21) the exchange of support for grooming is weaker	NA

1 This is in line with the model-based predictions by van Schaik and co-authors [128].

### Causation of coalition patterns in the model and predictions for empirical data

In empirical studies, patterns of reciprocation and exchange are considered to be based on record-keeping, so-called ‘calculated reciprocity’, if they remain statistically significant when proximity, rank, kinship and age are partialled out [20], [22], [30], as in this case they are not considered to be a side-effect of these factors [20], [91]. Unexpectedly, all the correlations for reciprocation and exchange in the model remain significant even when proximity and rank are partialled out (age and kinship are absent in the model, Tables S3). Thus, correlations in the model resemble empirical data. However, in the model, no records are kept by the individuals on acts given and received, nor on support or on grooming.

Because partial correlations may not sufficiently exclude the dynamics of rank and proximity [92], we did experiments in the model in which we removed the effects of rank and of proximity more rigorously than is achieved by partial correlation. We removed the effects of three different assumptions in turn, i.e., that interactions are influenced by social facilitation and by proximity (by making individuals choose interaction partners at random) and that there are differences among individuals in dominance rank (by shuffling ranks between adults). We investigated the consequences for the following eight patterns: percentage of coalitions, relative frequency of three coalition types, two patterns related to triadic awareness, and the occurrence of significance in four correlations (combined over 10 replica-runs), i.e., of reciprocation of support and opposition, grooming for receipt of support, and support for the receipt of grooming. The greatest reduction (i.e., 94%) in the number of significant patterns occurred when simultaneously disabling the effects of both proximity and rank, a slightly lower reduction occurred when merely disabling the effects of proximity, i.e., 50% at both intensities, a still lower reduction when omitting social facilitation (i.e., 50% at high intensity and 25% at low intensity) and when shuffling ranks, i.e., 38% at high intensity and 12% at low intensity (13–22 in Table 5).

**Table 5:** Dominance, affiliation and coalition patterns among females in the model when taking out different assumptions.

	A. No social facilitation		B. Ranks shuffled		C. Random interaction partners		D. Random interaction partners and ranks shuffled		E. Complete Model	
Intensity of Aggression	High	Low	High	Low	High	Low	High	Low	High	Low
**Dominance Style**										
1) Gradient of the hierarchy (CV)^1^	0.75	0.36	0.71	0.38	0.70	0.36	0.71	0.38	0.72	0.36
2) Unidirectionality of aggression (TauKr)	−0.19**	0.48***	**0.46** ***	0.53***	−0.54***	**−0.05**	**0.00**	**0.01**	−0.13**	0.51***
3) Time spent fighting (%)	14	20	16	20	NA	NA	NA	NA	13	17
4) Relative female dominance	0.29	0.00	**0.50**	**0.50**	0.16	0.00	0.50	**0.50**	0.22	0.00
5) Mean distance among all group members	26	22	**23**	**25**	NA	NA	NA	NA	29	25
6) Centrality of dominants (Tau)	−0.44***	−0.09	**0.06**	**−0.03**	NA	NA	NA	NA	−0.40**	−0.10
**Affiliative patterns**										
7) Time spent grooming (%)	18	22	**23**	**19**	NA	NA	NA	NA	17	20
8) Conciliatory Tendency	19	28	**32**	32	**0**	**0**	**0**	**0**	21	31
9) Grooming reciprocation (TauKr)	0.36***	0.51***	0.58***	0.55***	**−0.37** ***	2 **−0.01** **	**−0.03**	**−0.02**	0.39***	0.54***
10) Grooming up the hierarchy (TauKr)	0.34***	2 **0.05** *	**0.00**	0.00	0.50***	**0.14** **	0.09**	**0.09** **	0.34***	0.04
11) Grooming partners of similar ranks (TauKr)	0.17***	0.01	**0.00**	−0.03	0.07**	0.02	**−0.01**	−0.01	0.13**	−0.01
12) Reconciliation with valuable partners	0.36***	0.13**	0.15***	0.14**	**0.00**	**−0.02**	**−0.03**	**−0.03**	0.37***	0.11**
**Coalitions patterns**										
13) % of fights involving coalitions	**3%**	**3%**	9	9	**2%**	**1%**	**2**	**2**	10%	7%
14) Conservative coalitions %	52%	27%	**34%**	**32%**	64%	28%	**36%**	**34%**	71%	29%
15) Bridging coalitions %	37%	27%	**33%**	**34%**	23%	25%	**29%**	**32%**	21%	27%
16) Revolutionary coalitions %	11%	46%	**33%**	**34%**	13%	47%	**35%**	**34%**	8%	44%
Jonckheere-Terpstra test (C>B>R)	JT = 0***	JT = 240 NS	JT = 127 NS	JT = 167 NS	JT = 6***	JT = 220 NS	JT = 147 NS	JT = 155 NS	JT = 0***	JT = 205 NS
**Patterns related to triadic awareness**										
17) Recipient<Target<Supporter (%)^3^	+(74)***	−(25)***	**−(34)** ***	−(35)***	**+(62) NS**	−(23)***	**−(34)** ***	−(35)***	+(67)***	−(24)***
18) Support given to ‘friend’ (%)^3^	+(56)*	**+(54) NS**	**+(51) NS**	**+(54) NS**	+(77)***	+(54)*	**+(53) NS**	**+(57) NS**	+(70)***	+(54)*
**TauKr correlations**										
19) Support Reciprocation (TauKr)	2 **0.05** *	**0.08** **	0.21***	0.27***	2 **0.02** *	**0.04**	**0.00**	**0.01**	0.38***	0.27***
20) Grooming for Support Received (TauKr)	**0.23** ***	**0.18** ***	**0.26** ***	0.32***	**0.11** **	**−0.01**	**0.02**	**0.01**	0.36***	0.29***
21) Support for Grooming Received (TauKr)	**0.18** **	**0.23** ***	**0.33** ***	0.39***	**−0.01**	**−0.02**	**0.02**	**0.02**	0.29***	0.36***
22) Opposition given and opposition received	**0.00**	0.14**	**0.20** ***	0.29***	−0.07*	0.01*	**0.01**	**−0,03** **	−0.11**	0.29***

Results represent the average over 10 runs. P-value based on the Bonferroni correction:

* = p<0.05;

** = p<0.01,

*** = p<0.001. In **bold:** results that differ from the full model. NA = Not Available.

1 Among all individuals.

2 4 correlations (5% of 72 correlations) are considered to be a type I error.

3 Supporter higher ranking than target and recipient: + more frequent than chance; − less frequent than chance.

This led to the following explanations for the coalition patterns:

The *percentage of fights* that involved coalitions are a consequence of social facilitation and proximity, as can be seen from their decrease without these assumptions (13 in Table 5). Social facilitation strengthens the effects of proximity by increasing the likelihood of forming coalitions, because individuals that are close to a fight are activated next.

The *type of support* is a side-effect of risk aversion and individual differences in dominance rank, as can be seen when ranks are shuffled. In this case, the three types of support become similar in their frequency (14–16 in Table 5).

With reference to *triadic awareness* in the choice of coalition partners, the supporter is higher in rank than both the target and the receiver, as is the case for empirical data. However, in the model this is only found at high intensity of aggression and not at a low intensity (19 in Table 3). This pattern arises as a side-effect of rank and proximity, because it disappears if the effects of rank and space are removed (17B, 17C in Table 5). Clearly, individuals that are closer will have more opportunities to support each other and, at a high intensity, individuals that are of higher rank than an opponent and receiver will experience less risk in providing support. Since there are no data on triadic awareness among female primates in egalitarian species, we predict that in empirical studies on egalitarian species, females will also solicit others that are higher in rank less often than both the solicitor and target, than is the case in despotic species (18 in Table 4).

*Reciprocation of support* among females is due to social facilitation and proximity. This is clear, because it is weakened when social facilitation is disabled and it disappears after taking out proximity and making individuals randomly choose interaction partners (19AC in Table 5). Reciprocation of support emerges because certain individuals are more often in close proximity than other individuals and, thus have more opportunities for attacking the same opponents. In fact, two individuals may attack the same target in turn for several consecutive activations when the victim, by fleeing from one opponent, ends up in the space occupied by the other opponent, a kind of spatial entrapment (see video S1) [93]. Such immediate reciprocation happens at high intensity in 25% of the cases of support and at low intensity in 7% of cases. When we exclude immediate reciprocation, the patterns in Table 3 remain, but the percentage of fights involving coalitions decreases at high intensity of aggression (from 10 to 7%, 1 in Table S4), and reciprocation of support is weakened at both intensities, but still significant in all runs (5 in Table S4). Further, the interchange of grooming for receipt of support and of support for receipt of grooming remains similar in significance without immediate reciprocation (6,7 in Table S4). This interchange emerges as a side-effect of proximity and rank: these correlations are significantly weakened when the effects of social facilitation and proximity are excluded and become non-significant if females choose their interaction partners at random and their ranks are simultaneously shuffled (20, 21 in Table 5).

*Opposition* in the model is bidirectional at low intensity of aggression (thus, individuals more often oppose those partners from whom they receive more opposition [87]) and unidirectional at high intensity of aggression (25 in Table 3). This also applies if we exclude immediate reciprocation (8 in Table S4). This is expected, as no separate rule for support (or opposition) has been added (both are in the eye of the observer), opposition *is* a specific instance of dyadic aggression, and dyadic aggression is more bidirectional at low than at high intensity of aggression (2 in Table 3) [36]–[38]. Furthermore, as expected, opposition is significantly correlated with the remaining cases of dyadic aggression (6, 7 in Table S2). Patterns of bidirectionality at low intensity of aggression and unidirectionality at high intensity disappear after taking out both spatial structure and the effects of ranks by shuffling ranks (22D in Table 5).

Correlations for reciprocation of opposition and for opposition with grooming and support remain when immediate reciprocation is excluded, Table S4. They are a side-effect of correlations for dyadic aggression with grooming and support (8–12 in Table S2). The patterns of bi- and unidirectionality of opposition, correlations for opposition with aggression and for ‘exchange’ between opposition and support or grooming may be used directly as model-based predictions to be tested empirically (7–12, 13, 15 in Table 4).

### Differences between high and low intensity

Regarding patterns indicating triadic awareness in the choice of coalition partner, supporters are more often higher ranked than the target and the receivers at high intensity compared to low intensity, because due to the steep hierarchy, supporters of lower rank experience more risk of being defeated, whereas such risks for individuals of different ranks are more similar at low intensity due to the weak hierarchy.

With reference to the *type of coalitions among females*, the percentage of conservative coalitions is higher at high aggression intensity, as a consequence of the hierarchy being steeper than at low intensity (1 in Table 3). The steeper hierarchy increases the aversion of attacking higher ranking individuals and the likelihood of attacking lower ranking individuals, thus leading to conservative coalitions most often, and to bridging coalitions at an intermediate frequency (16–18 Table 3). In contrast, revolutionary coalitions between females are more frequent at low intensity of aggression. This is due to the weaker hierarchy and the stronger subordinance of females to males at a low aggression intensity than at a high aggression intensity (4 in Table 3), which resembles the greater subordinance of female egalitarian macaques to males compared to despotic macaques [42]. Indeed, when we exclude coalitions of females against males at a low intensity, revolutionary coalitions become less frequent than bridging and conservative coalitions, as is the case for high intensity, C>B>R (Jonckheere-Terpstra test, n = 10, JT = 28.5, p<0.001). At low intensity, the number of opportunities for females to attack higher ranking individuals is greater than at high intensity for two reasons: 1) the subordinance of females relative to males is greater than at high intensity (4 in Table 3) and 2) the percentage of males in the group is higher (30% vs. 20% at high intensity). With reference to the percentage of males, if the percentage of males in the group is increased from approximately 25% (20% at high intensity or 30% at low intensity), via 50% to 70% in the model, the number of revolutionary coalitions among females increases from 8 to 10 to 20% at high intensity and from 44 to 55 to 73% at low intensity. Thus, we predict that the higher the percentage of males in the group, the higher the frequency of revolutionary coalitions compared to conservative or bridging coalitions (1 in Table 4). Because empirical data on coalition types in egalitarian species are lacking, this result serves as a prediction: coalitions among females in egalitarian species should more often be revolutionary than in despotic species (17 in table 4).

At a high intensity, females *reciprocate support* more often than at a low intensity, because reciprocation more often happens immediately. This is because individuals in the group are spaced further apart (5 in Table 3) and series of immediate reciprocation thus continue for longer because there is less ‘interference’ from other individuals close by. The greater spacing of individuals in the group is a consequence of the repeated fleeing of lower ranking individuals, due to the steeper hierarchy [38]. Because the spacing of individuals in groups of despotic macaques is also greater than that in egalitarian macaques, we predict that empirical data for despotic societies compared to egalitarian societies will reveal relatively less frequent revolutionary support and conservative support to be more frequent, and support to be reciprocated more often (17,19 in Table 4). Furthermore, there is a stronger correlation for exchange of grooming for support and a weaker correlation for support for grooming at a high intensity than at a low intensity (20, 21 in Table 4; 2, 3 in Table S3). This is a consequence of the fact that at high intensity, both variables, grooming and receipt of support, are significantly positively correlated with the rank of the partner (10 in Table 3; 3 in Table S2), while this is not the case for the variables of support and receipt of grooming (4, 5 in Table S2). This results in the model-based prediction for high intensity, that individuals receive support more frequently from partners, the higher the rank of the partners, for which there is also some empirical evidence (14 in Table 4). Other patterns, such as the association between grooming other individuals and supporting them (1,2 in table S2), can also be used as hypotheses for empirical data (5,6 in Table 4).

### Sensitivity-analysis of coalition patterns

The patterns of reciprocation and exchange appear to be robust against changes to the parameters, as they depend only weakly on the percentage of coalitions, the number of individuals and the degree of aversion of risks. They remain significant as long as the percentage of coalitions is above ∼4% for females (see caption in Table S5) and the number of females is at least 8 at high and 12 at low intensity of aggression (Table S5). If the risk aversion is increased from winning twice mentally before attacking to winning mentally 3, 4 or 5 times, the patterns of types of support, exchange and reciprocation of support and opposition remain qualitatively the same (Table S5).

The patterns of reciprocation of support and its exchange for grooming also appear to be robust against changes in the behavioural rules. They appear to remain significant under the following experimental manipulations (Text S1 and Table S1): 1) when we change the order of the rules for aggression and grooming (by reversing the order, by first considering grooming and then fighting and by taking a random order in which to consider both acts, column AB in Table S1), 2) when we omit the induction of grooming by anxiety and instead make individuals always groom when they expect to lose a fight (C in table S1), and 3) when omitting the aversion of the risk of losing a fight, but giving individuals a specific chance of attacking at high intensity and at low intensity (see experimental setup), independent of the risks involved (column D in table S1). The proportions of different types of coalitions only changed compared to the full model when risk-aversion at high intensity was omitted (Table S1). Note that the manipulation of omitting risk aversion is similar to shuffling ranks. With reference to reciprocation (bidirectionality) of opposition, unidirectional opposition at high intensity depends on risk aversion and on the order of the behavioural rules in the same way as dyadic aggression (22 in columns A and D in Table S1). Patterns that may be considered indications of triadic awareness in the choice of coalition partners depend on risk aversion and on the order of the behavioural rules at high aggression intensity (17, 18 in Table S1).

## Discussion

We have shown that our model does a good job at predicting the relative percentage of different types of coalitions, patterns indicative of triadic awareness in the choice of coalition partners and patterns of reciprocation and exchange. The model succeeds at this by reducing the problem to the right variables. It reveals how patterns of support and opposition, their reciprocation and exchange may emerge as a side-effect of socio-spatial structure through self-organization. The processes of socio-spatial structuring are mostly a consequence of dominance interactions [37], [38]. Rank-related patterns (such as more frequent grooming of other individuals higher in rank at high intensity of aggression) are due to rank and aversion of the risks of being defeated [36]. Patterns of support are due to socio-spatial structure, with social facilitation playing a lesser role. These patterns arise because the socio-spatial structure implies that certain individuals are often close to specific other individuals. This automatically causes the occurrence of support (and opposition) in fights, reciprocation and exchange for grooming. The experiments in the model and the sensitivity analysis of its parameters and behavioural rules show that the occurrence of support, its reciprocation and exchange are robust. This is surprising, because the model drops ‘rational’ or ‘deliberate’ choices by individuals to support others in fights, it lacks triadic awareness and lacks record-keeping. Similar processes of socio-spatial structuring through dominance interactions and differences in fighting power (rank) and avoidance of risks, may also automatically induce patterns of support and opposition, their reciprocation and exchange in real primates. Indeed spatial centrality of dominants is also found in real primates [94]–[102] and seems stronger in despotic species than in egalitarian species [38].

It is worth comparing existing explanations of a number of empirical findings to those of the present model.

First, the finding that chimpanzees reciprocated both support and opposition and that macaques reciprocated only support but not opposition has been taken as evidence that the chimpanzees simultaneously consider more aspects of social relationships than macaques and that chimpanzees are revengeful, but macaques are not [20], [34]. However, no reciprocation of opposition was found for chimpanzees in the same data set when data were analyzed on an annual basis (instead of being lumped over five consecutive summers), neither was opposition reciprocated when studied by sex [30]. Absence of reciprocation of opposition is in line with the model because reciprocation of opposition is absent at high intensity and we assume that chimpanzees in this colony are despotic rather than egalitarian, because the dominance style of chimpanzees is most despotic in communities (such as Ta�) where grouping is densest [103]. In this captive colony, grouping is dense and frequency of aggression is high as well, which results in a more despotic dominance style than when the individuals in groups are more spaced apart and aggression is rarer, as is the case in natural conditions [104]. Despotism in this captive colony is also apparent because the higher the rank of the partner, the more often the females in this colony groom others [30], which is a pattern that is typical of macaques that are despotic, but not of those that are egalitarian [105].Thus, lack of reciprocation of opposition in the Arnhem colony is in line with the model, which suggests that reciprocation of opposition is constrained by avoidance of the risks of attacking higher ranking individuals because the hierarchy is steep. In contrast, if the hierarchy is weak, opposition automatically becomes more reciprocal (also referred to as bi-directional), because the mutual risks are more similar. Thus, the model offers up the difference in the hierarchical gradient as an alternative explanation to the usually assumed difference in intelligence.

Second, Silk [45] finds reciprocation of support and opposition in male bonnet macaques (*Macaca radiata*). This is also in line with the model as bonnet macaques are egalitarian [51]. Silk reasons that if individuals classify other individuals into allies and adversaries, they should more often give support to those whom they oppose less. Contrary to this, her data show instead that individuals more frequently support those individuals that they oppose more often. This association reflects what our model predicts.

Third, stronger patterns of coalition formation have been found in despotic than egalitarian species among female macaques and this has been attributed to the stronger effects of kin and nepotism [106]. Although this may be true, our model indicates possible alternative causes. It suggests that stronger reciprocation of support among despotic females than among egalitarian females is due to the higher degree of immediate reciprocation, which is caused by the greater spacing between females in the despotic group. The lower spatial density in the model lengthens the chains of mutual support in fights that are undisturbed [92].

Fourth, empirical data reveal that individuals solicit support by headflagging more often to other individuals ranked above them and to those with better relationships with themselves than with the opponent [13], [15], [17], [23]. In the model, although headflagging is absent, individuals still receive more support from higher ranking individuals, but this is not due to triadic awareness in the model. Instead, it arises as a side-effect of rank and proximity (17 in Table 5). Individuals may also be more easily solicited in reality when they are closer to the solicitor and the fight. Those individuals that are closer to the solicitor are the individuals that experience less risk, thus, they will be the individuals that are higher ranked than the other two (i.e., the potential receiver and the target).

Fifth, in several species, individuals more often support those individuals in fights that they also groom more frequently [30], [107]. This has been explained by cognitive mechanisms, but classical conditioning has also been suggested [30], [108]. The present model provides an even simpler explanation, the association is a side-effect of spatial proximity.

Sixth, when patterns of reciprocation and exchange remain significant, after partialling out proximity, kinship, rank and age, it is concluded that reciprocation and exchange are ‘calculated’ by record-keeping [20], [22], [30]. However, these are not calculated in the model and patterns of reciprocation and exchange still remain after partialling out rank and proximity. Apparently, these statistical procedures do not deal satisfactorily with complex nonlinear effects due to the socio-spatial structuring [11], [37], [92], because when we remove the effects of proximity (or both rank and proximity) by an experimental procedure in the model, reciprocation and exchange are no longer significant (19–22CD in Table 5). Thus, the model shows that it does not suffice to partial out proximity in order to eliminate its effects. It appears that the partial correlation has not completely excluded the dynamics of these effects because a partial correlation represents a linear, additive approach and effects of fights on spatial structure are nonlinear [109]. This serves as an important warning for the interpretation of these correlations.

Two important features of our work, its parsimony and falsifiability, are reached by integrating many aspects, such as spatial position, fights and grooming. As a consequence, the model produces explanations that are cognitively parsimonious and hypotheses that are easily tested because they concern aspects on which much empirical data are available, such as dominance style [38], [42], [110], affiliative behaviour [36] and coalitions in egalitarian and despotic societies (Table 4).

Due to the repeated process of validation of our model over a decade, we have gained more and more confidence in it [111]; first, we have shown that the patterns of the model at low and high intensity of aggression resemble, respectively, egalitarian and despotic societies regarding dominance style (namely, frequency of aggression, average distance between individuals, symmetry of aggression, spatial centrality of dominants, and decrease of aggression when becoming ‘familiarized’) [37], [38], [85]; second, we have predicted and confirmed greater female dominance relative to males when dominance style is steeper and when the percentage of males in the group is higher [42]; third, we have shown that adding a rule of intending to groom to avoid the risks of losing a fight and when being anxious led to patterns of grooming and reconciliation resembling empirical data for both dominance styles in macaques [36]; fourth, in the present paper, we show that the model also reveals patterns of support (and opposition), reciprocation and interchange for grooming that resemble those in real primates.

A point of critique by de Vries on an earlier study of our model [112] has been that the directional inconsistency of the dominance interactions is too low compared to that found in empirical data. Due to the increased risk aversion in the current model (but for the same number of fights), directional inconsistency has become higher (0.91 among adults at a high intensity), while qualitatively maintaining all reported results (Table S5) [38]. This value resembles that found in empirical data on despotic macaques, *M. fuscata* and *M. fascicularis* (Table 2 of de Vries). Whether the directional inconsistency characterizes dominance style in a useful way is, however, doubtful, because de Vries shows it to be higher in egalitarian macaques than in despotic macaques [112], whereas we would expect the opposite to hold.

In the present study, the frequency of polyadic fights is lower than in reality. Note that the model presented here was constructed before looking at data on coalitions. Instead, it was loosely tuned to grouping and aspects of dominance style and percentage of grooming [36]. The frequency of polyadic coalitions may be heightened by increasing the biological realism of the model, e.g., by including sexual behaviour. When we add sexual attraction of males to females and make females come into oestrus asynchronously, males have been shown to cluster close to a female in oestrus [80]. Therefore, we may expect a higher number of polyadic coalitions among these males [113].

The model is an extreme simplification of reality. Its social complexity and biological realism could be increased, e.g., by including recruitment behaviour, social bonding, feeding behaviour, kin-relations, different sex-age classes, immigration or emigration or sexual behaviour. It should be stressed that our model is not meant to show that primates are unintelligent. That primates are intelligent is proven, for instance by the fact that they show intentional imitation [114] and intentional exchanges in experimental settings [26]. For some species, the model may represent coalitions as they are at present, but for others they may represent coalitionary behaviour as it was early in evolution, because coalitions of these species have recently become cognitively more sophisticated. In future, we will also use models to study more sophisticated cognitive strategies of supporting others in fights. However, it should be noted that even if primates are using more intelligent strategies for coalitions, there will still be an effect of socio-spatial structure on coalition patterns.

With regard to evolutionary explanations, our model indicates that selection operates on complexes of interconnected traits rather than single traits alone. For instance, according to our model, the evolution of a higher intensity of aggression versus a milder intensity is associated with automatic consequences for many traits, e.g., a steeper hierarchy, greater female dominance over males, less reconciliation, fewer revolutionary coalitions, stronger reciprocation and exchange of support. Therefore, theories will need to explain the evolution of the whole complex of integrated traits. To relate the evolution of this complex to ecological conditions, models must examine its evolution for several distributions of food in a similar way as has been done in models related to culture [115], [116].

Our results have three clear implications. First, in contrast to the common belief in empirical studies [20], [22], [30], correlations for reciprocity and exchange in the model remain significant after partialling out proximity and rank, even though these correlations are not due to intentional or internally-guided rules for reciprocation or exchange. A different method other than partial correlation is apparently required to exclude the effects of proximity and of rank in the causation of patterns of reciprocation and exchange. Secondly, for scientists interested in the actual cognitive deliberation underlying spontaneous social behaviour in groups of primates, it is essential to study the spatial positioning of individuals in relation to their social behaviour. This is necessary in order to see to what extent the social behaviour can be attributed to socio-spatial structuring and what patterns are left that must be attributed to active deliberation. Thirdly, this model presents a starting point for developing a theory of social behaviour that arises among individuals if only simple cognition is present. Such theories are badly needed [104], [117].

## Supporting Information

Figure S1 **Rules of behavioural interaction.** White boxes: grouping rules, black boxes: rules for dominance interactions, and light grey boxes: rules of affiliation.
(TIF)

Table S1 **Sensitivity analysis of the behavioural rules.** Patterns among females. Results represent the average over 10 runs; P-value based on the Bonferroni correction: *p = <0.05; **p = <0.01, ***p = <0.001. In **bold:** results that differ from the full model. ^1^Supporter higher ranking than target and recipient: + more frequent than chance; − less frequent than chance.
(DOC)

Table S2 **TauKr correlations concerning grooming, support, opposition and rank among females in GrooFiWorld.** Results represent the average TauKr value of 10 runs; Significance is based on the Bonferroni correction: * = p<0.05; ** = p<0.01, *** = p<0.001. ^1^1 correlation (5% of 24 correlations) is considered to be a type I error.
(DOC)

Table S3 **Tau Kr correlations for reciprocation and exchange of support among females in GrooFiWorld when partialling out rank and proximity.** Values represent the average over 10 runs; In **bold:** results that differ significantly from the non-partial correlation. P-value based on the Bonferroni correction: *p = <0.05; **p = <0.01, ***p = <0.001.^†^ MW U test = Mann-Whitney U test between high and low intensity of aggression, H = higher at high intensity of aggression; L = higher at low intensity of aggression; NS = not significant; n_1_ = 10, n_2_ = 10.
(DOC)

Table S4 **Coalition patterns after controlling for immediate reciprocity.** Patterns among females. Results represent the average over 10 runs; P-value based on the Bonferroni correction: *p = <0.05; **p = <0.01, ***p = <0.001.
(DOC)

Table S5 **Sensitivity analysis of parameters of the complete model.** Patterns among females in GrooFiWorld for several group sizes, sex ratios and degrees of risk aversion. Results represent the average over 10 runs; P-value based on the Bonferroni correction: *p = <0.05; **p = <0.01, ***p = <0.001. In **bold:** results that differ from the full model (in Table 3). ^1^At low intensity of aggression a risk aversion base of 5 implies an actual number of decisions to avoid risks of ∼1 (see table 2). ^2^Among all individuals. ^3^One correlation (5% of 24 correlations) is considered to be a type I error. ^4^For a social facilitation of 10%, the percentage of fights involving coalitions is ∼4% and all coalition patterns among females are retained (results not shown, data available on request).
(DOC)

Text S1 **Description of the details of the model and of extra results that have been mentioned and discussed in the main text.**
(DOC)

Video S1 **Example of coalitions in GrooFiWorld.** In this video we show three individuals fighting (triadic interaction). The individuals are represented by circles and their directions of movement by blue arrows. The winner of the fight turns red and the loser blue. Several instances of immediate reciprocation of support can be observed. The two individuals on the top-right and bottom-left of the screen alternate in attacking the individual in the middle. Thus, the cooperators immediately reciprocate each other's support.
(AVI)
