# Supplementary figures and images for: An Individual-Oriented Model on the Emergence of Support in Fights, Its Reciprocation and Exchange
Source: PLoS One. 2012 May 30;7(5):e37271. doi: 10.1371/journal.pone.0037271 (PMC3364247; doi:10.1371/journal.pone.0037271)

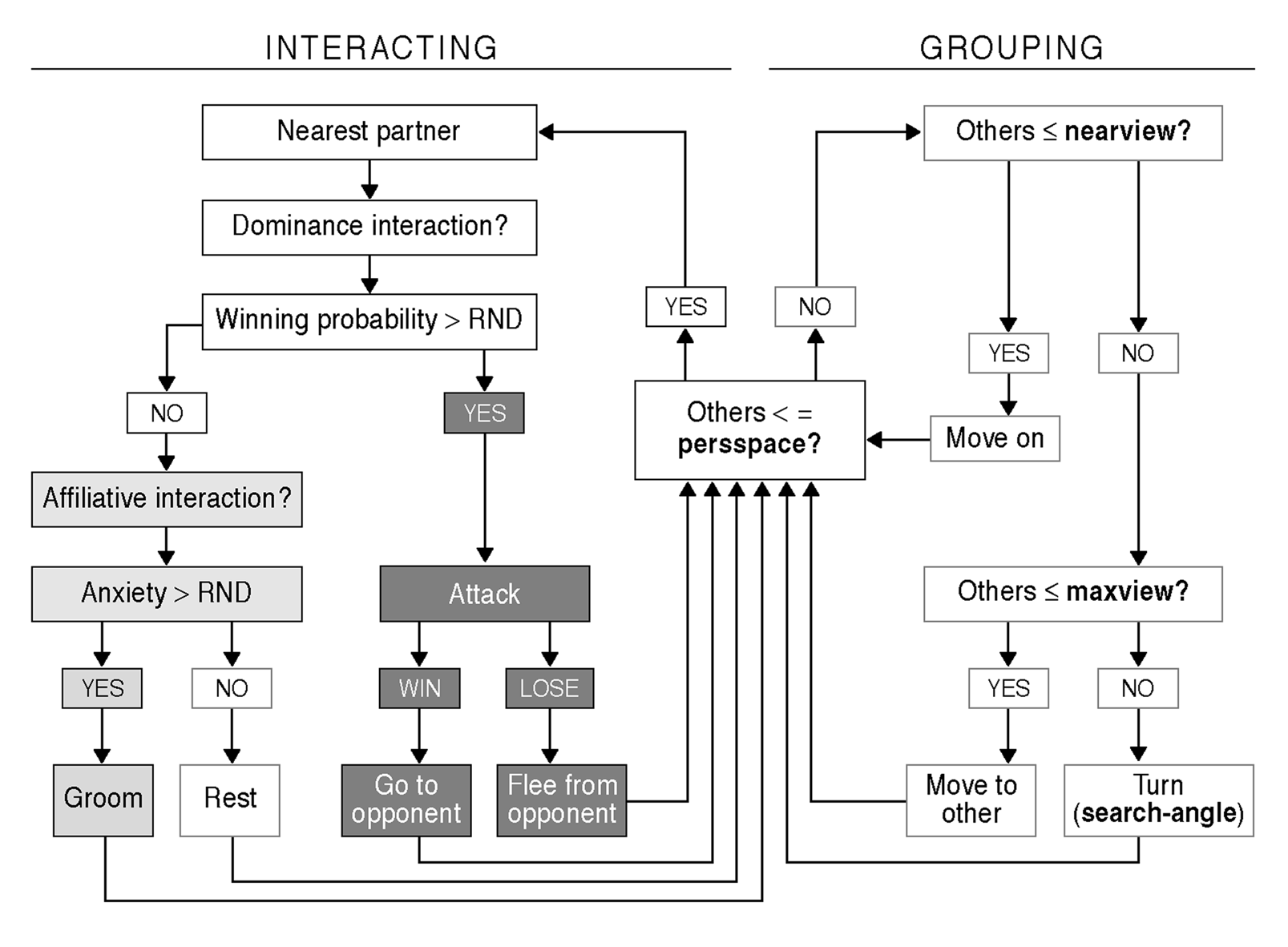

Supplement: Figure S1 — Rules of behavioural interaction. White boxes: grouping rules, black boxes: rules for dominance interactions, and light grey boxes: rules of affiliation. (TIF) [file pone.0037271.s001.tif]
