# Supplementary material for: An Individual-Oriented Model on the Emergence of Support in Fights, Its Reciprocation and Exchange
Source: PLoS One. 2012 May 30;7(5):e37271. doi: 10.1371/journal.pone.0037271 (PMC3364247; doi:10.1371/journal.pone.0037271)
Supplement: Table S1 — Sensitivity analysis of the behavioural rules. Patterns among females. Results represent the average over 10 runs; P-value based on the Bonferroni correction: *p = <0.05; **p = <0.01, ***p = <0.001. In bold: results that differ from the full model. 1Supporter higher ranking than target and recipient: + more frequent than chance; − less frequent than chance. (DOC) [file pone.0037271.s002.doc]

**Table S1. Sensitivity analysis of the behavioural rules.**

| Behavioural Rules | **A)** First considering grooming; then, fighting | | **B)** Random order of considering grooming and fighting | | **C)** No anxiety-induced grooming | | **D)** No Risk Aversion | | **E)** GrooFiWorld Complete Model | |
| --- | --- | --- | --- | --- | --- | --- | --- | --- | --- | --- |
| Intensity of Aggression | High | Low | High | Low | High | Low | High | Low | High | Low |
| **Dominance Style** |  |  |  |  |  |  |  |  |  |  |
| 1) Gradient of the hierarchy (CV)^1^ | **0.54** | 0.36 | 0.62 | 0.37 | 0.69 | 0.38 | 0.71 | 0.36 | 0.72 | 0.36 |
| 2) Unidirectionality of aggression (TauKr) | **0.03** | 0.32*** | **0.09**** | 0.49*** | -0.11* | 0.52*** | **0.45***** | 0.50*** | -0.13** | 0.51*** |
| 3) Time spent fighting (%) | **5** | **6** | 7 | 10 | 12 | 16 | 13 | 17 | 13 % | 17 % |
| 4) Relative female dominance | 0.11 | 0.00 | 0.15 | 0.0 | 0.23 | 0.00 | 0.21 | 0.00 | 0.22 | 0.00 |
| 5) Mean distance among all group members | 23 | 23 | 30 | 26 | 27 | 25 | **24** | **26** | 29 | 25 |
| 6) Centrality of dominants (Tau) | **-0.07** | **0.02** | -0.29* | -0.12 | -0.36** | -0.04 | -0.26** | -0.03 | -0.40** | -0.10 |
| **Affiliative patterns** |  |  |  |  |  |  |  |  |  |  |
| 7) Time spent grooming (%) | **37** | **38** | 20 | 23 | 19 | 21 | **27** | 20 | 17 | 20 |
| 9) Grooming reciprocation (TauKr) | 0.67*** | 0.67*** | 0.71*** | 0.71*** | 0.40*** | 0.55*** | 0.62*** | 0.60*** | 0.39*** | 0.54*** |
| 10) Grooming up the hierarchy (TauKr) | **0.00** | 0.00 | **0.00** | 0.02 | 0.32*** | 0.03 | **0.00** | 0.02 | 0.34*** | 0.04 |
| 11) Grooming partners of similar ranks (TauKr) | **0.01** | 0.00 | **0.02** | 0.02 | 0.12** | 0.02 | **-0.01** | 0.00 | 0.13** | -0.01 |
| **Coalitions patterns** |  |  |  |  |  |  |  |  |  |  |
| 13) % of fights involving coalitions | **2** | **3** | **4** | **4** | 10 | 7 | **6** | 7 | 10 | 7 |
| 14) Conservative coalitions % | 57 | 27 | 66 | 29 | 74 | 28 | **31** | 23 | 71 | 29 |
| 15) Bridging coalitions % | 26 | 24 | 21 | 25 | 18 | 27 | **31** | 23 | 21 | 27 |
| 16) Revolutionary coalitions % | 17 | 49 | 13 | 46 | 8 | 45 | **38** | 54 | 8 | 44 |
| **Patterns related to triadic awareness** |  |  |  |  |  |  |  |  |  |  |
| 17) Recipient < Target < Supporter (%)^1^ | **-(49)NS** | -(23)*** | **+(55)NS** | -(24)*** | +(64)*** | -(24)%*** | **-(28)***** | -(17)*** | +(67)*** | -(24)*** |
| 18) Support given to ‘friend’ (%)^1^ | **+(52)NS** | **+(52)NS** | **+(58)NS** | **+(53)NS** | +(72)*** | +(53)%* | **+(53)NS** | +(55)* | (+70)*** | +(54)* |
| **TauKr correlations** |  |  |  |  |  |  |  |  |  |  |
| 19) Support Reciprocation (TauKr) | 0.15* | 0.08** | 0.17** | 0.13** | 0.41*** | 0.27*** | 0.16** | 0.17** | 0.38*** | 0.27*** |
| 20) Grooming for Support Received (TauKr) | 0.10** | 0.13** | 0.14** | 0.19*** | 0.36*** | 0.28*** | 0.22*** | 0.25*** | 0.36*** | 0.29*** |
| 21) Support for Grooming Received (TauKr) | 0.13** | 0.16** | 0.18** | 0.23*** | 0.30*** | 0.35*** | 0.27*** | 0.33*** | 0.29*** | 0.36*** |
| 22) Opposition given and opposition received | **0.01** | **0.00** | -0.03* | 0.11** | -0.07** | 0.25*** | **0.12**** | 0.20*** | -0.11** | 0.29*** |

Patterns among females. Results represent the average over 10 runs; P-value based on the Bonferroni correction: *p=<0.05; **p=<0.01, ***p=<0.001. In **bold:** results that differ from the full model. ^1^Supporter higher ranking than target and recipient: + more frequent than chance; - less frequent than chance.
