# Supplementary material for: An Individual-Oriented Model on the Emergence of Support in Fights, Its Reciprocation and Exchange
Source: PLoS One. 2012 May 30;7(5):e37271. doi: 10.1371/journal.pone.0037271 (PMC3364247; doi:10.1371/journal.pone.0037271)
Supplement: Table S2 — TauKr correlations concerning grooming, support, opposition and rank among females in GrooFiWorld. Results represent the average TauKr value of 10 runs; Significance is based on the Bonferroni correction: * = p<0.05; ** = p<0.01, *** = p<0.001. 11 correlation (5% of 24 correlations) is considered to be a type I error. (DOC) [file pone.0037271.s003.doc]

**Table S2. TauKr correlations concerning grooming, support, opposition and rank among females in GrooFiWorld.**

| Intensity of Aggression | High | Low |
| --- | --- | --- |
| 1) Grooming and Support | 0.25*** | 0.35*** |
| 2) Receipt of grooming and receipt of support | 0.19** | 0.28*** |
| 3) Receipt of support and rank of partner | 0.26*** | 0.14 |
| 4) Receipt of grooming and rank of partner | ^1^-0.05* | -0.02 |
| 5) Support and rank of partner | 0.06* | 0.00 |
| 6) Aggression and opposition | 0.52*** | 0.43*** |
| 7) Receipt of aggression and receipt of opposition | 0.47*** | 0.33*** |
| 8a) Opposition and Opposition Received | -0.11** | 0.29*** |
| 8b) Aggression and aggression received | -0.11** | 0.48*** |
| 9a) Grooming and Opposition Received | 0.43*** | 0.35*** |
| 9b) Grooming and aggression received | 0.63*** | 0.53*** |
| 10a) Opposition and Grooming Received | 0.31*** | 0.41*** |
| 10b) Aggression and grooming received | 0.46*** | 0.53*** |
| 11a) Support and Opposition | 0.13** | 0.28*** |
| 11b) Support and aggression | 0.20*** | 0.37*** |
| 12a) Support and Opposition Received | 0.20*** | 0.27*** |
| 12b) Support and aggression received | 0.23*** | 0.37*** |

Results represent the average TauKr value of 10 runs; Significance is based on the Bonferroni correction: *=p<0.05; **=p<0.01, ***=p<0.001. ^1^1 correlation (5% of 24 correlations) is considered to be a type I error.
