# Supplementary material for: An Individual-Oriented Model on the Emergence of Support in Fights, Its Reciprocation and Exchange
Source: PLoS One. 2012 May 30;7(5):e37271. doi: 10.1371/journal.pone.0037271 (PMC3364247; doi:10.1371/journal.pone.0037271)
Supplement: Table S3 — Tau Kr correlations for reciprocation and exchange of support among females in GrooFiWorld when partialling out rank and proximity. Values represent the average over 10 runs; In bold: results that differ significantly from the non-partial correlation. P-value based on the Bonferroni correction: *p = <0.05; **p = <0.01, ***p = <0.001.† MW U test = Mann-Whitney U test between high and low intensity of aggression, H = higher at high intensity of aggression; L = higher at low intensity of aggression; NS = not significant; n1 = 10, n2 = 10. (DOC) [file pone.0037271.s004.doc]

**Table S3.** Tau Kr correlations for reciprocation and exchange of support among females in GrooFiWorld when partialling out rank and proximity.

|  | GrooFiWorld | | ^†^MW U test |
| --- | --- | --- | --- |
| Intensity of aggression | High | Low |  |
| 1) Support Reciprocation | 0.38*** | 0.27*** | H 95*** |
| 1a) Rank partialled out | 0.37*** | 0.27** | H 94*** |
| 1b) Proximity partialled out | **0.32***** | **0.20***** | H 95*** |
| 2) Grooming for Support Received | 0.36*** | 0.29*** | H 95*** |
| 2a) Rank partialled out | **0.30***** | 0.29*** | H 58 NS |
| 2b) Proximity partialled out | **0.26***** | **0.21***** | H 83* |
| 3) Support for Grooming Received | 0.29*** | 0.36*** | L 85** |
| 3a) Rank partialled out | 0.29*** | 0.36*** | L 84** |
| 3b) Proximity partialled out | **0.21***** | **0.27***** | L 80* |
| 4) Opposition reciprocation | -0.11** | 0.29*** | L 100*** |
| 4a) Rank partialled out | **0.00** | 0.29*** | L 100*** |
| 4b) Proximity partialled out | -0.14** | **0.22***** | L 100*** |

Values represent the average over 10 runs; In **bold:** results that differ significantly from the non-partial correlation. P-value based on the Bonferroni correction: *p=<0.05; **p=<0.01, ***p=<0.001.^†^ MW U test = Mann-Whitney U test between high and low intensity of aggression , H= higher at high intensity of aggression; L = higher at low intensity of aggression; NS= not significant; n_1_=10, n_2_=10.
