# Supplementary material for: An Individual-Oriented Model on the Emergence of Support in Fights, Its Reciprocation and Exchange
Source: PLoS One. 2012 May 30;7(5):e37271. doi: 10.1371/journal.pone.0037271 (PMC3364247; doi:10.1371/journal.pone.0037271)
Supplement: Table S4 — Coalition patterns after controlling for immediate reciprocity. Patterns among females. Results represent the average over 10 runs; P-value based on the Bonferroni correction: *p = <0.05; **p = <0.01, ***p = <0.001. (DOC) [file pone.0037271.s005.doc]

**Table S4**. Coalition patterns after controlling for immediate reciprocity.

|  | GrooFiWorld | | GrooFiWorld without immediate reciprocation | |
| --- | --- | --- | --- | --- |
| Intensity of Aggression | High | Low | High | Low |
| 1) % of fights involving coalitions | 10% | 7% | 7% | 7% |
| 2) Conservative coalitions % | 71% | 29% | 64% | 28% |
| 3) Bridging coalitions % | 21% | 27% | 26% | 27% |
| 4) Revolutionary coalitions % | 8% | 44% | 10% | 45% |
| 5) Reciprocation of support (TauKr) | 0.38*** | 0.27*** | 0.18*** | 0.20*** |
| 6) Grooming for Support Received (TauKr) | 0.36*** | 0.29*** | 0.35*** | 0.28*** |
| 7) Support for Grooming Received (TauKr) | 0.29*** | 0.36*** | 0.29*** | 0.35*** |
| 8) Opposition given and opposition received | -0.11** | 0.29*** | -0.07** | 0.28*** |
| 9) Opposition given and grooming received | 0.31*** | 0.41*** | 0.34*** | 0.40*** |
| 10) Grooming given and opposition received | 0.43*** | 0.35*** | 0.43*** | 0.34*** |
| 11) Support given and opposition given | 0.13*** | 0.28*** | 0.17** | 0.26*** |
| 12) Support given and opposition received | 0.20*** | 0.27*** | 0.19*** | 0.26*** |

Patterns among females. Results represent the average over 10 runs; P-value based on the Bonferroni correction: *p=<0.05; **p=<0.01, ***p=<0.001.
