# Supplementary material for: An Individual-Oriented Model on the Emergence of Support in Fights, Its Reciprocation and Exchange
Source: PLoS One. 2012 May 30;7(5):e37271. doi: 10.1371/journal.pone.0037271 (PMC3364247; doi:10.1371/journal.pone.0037271)
Supplement: Table S5 — Sensitivity analysis of parameters of the complete model. Patterns among females in GrooFiWorld for several group sizes, sex ratios and degrees of risk aversion. Results represent the average over 10 runs; P-value based on the Bonferroni correction: *p = <0.05; **p = <0.01, ***p = <0.001. In bold: results that differ from the full model (in Table 3). 1At low intensity of aggression a risk aversion base of 5 implies an actual number of decisions to avoid risks of ∼1 (see table 2). 2Among all individuals. 3One correlation (5% of 24 correlations) is considered to be a type I error. 4For a social facilitation of 10%, the percentage of fights involving coalitions is ∼4% and all coalition patterns among females are retained (results not shown, data available on request). (DOC) [file pone.0037271.s006.doc]

**Table S5. Sensitivity analysis of parameters of the complete model.**

| Sex | Females | Females | Females |
| --- | --- | --- | --- |
| Intensity of Aggression | High | Low | ^1^High |
| Number of females; males | 8;2 | 12;5 | 24;6 |
| Base of risk aversion; number of actual decisions | 2; ~2 | 2; ~1 | 5; ~5 |
| **Dominance Style** |  |  |  |
| 1) Gradient of the hierarchy (CV)^2^ | 0.81 | 0.37 | 0.71 |
| 2) Unidirectionality of aggression (TauKr) | -0.44* | 0.46*** | -0.45*** |
| 3) Time spent fighting (%) | 14 | 18 | 9 |
| 4) Relative female (male) dominance | 0.17 | 0.00 | 0.17 |
| 5) Mean distance among all group members | 16 | 18 | 24 |
| 6) Centrality of dominants (Tau) | -0.59* | -0.03 | -0.47*** |
| **Affiliative patterns** |  |  |  |
| 7) Time spent grooming (%) | 16 | 20 | 26 |
| 8) Conciliatory Tendency | 15 | 30 | 14 |
| 9) Grooming reciprocation (TauKr) | 0.35** | 0.48*** | 0.57*** |
| 10) Grooming up the hierarchy (TauKr) | 0.45** | **^3^0.10*** | 0.29*** |
| 11) Grooming partners of similar ranks (TauKr) | 0.31* | -0.11 | 0.23*** |
| 12) Reconciliation with valuable partners | 0.44** | 0.02 | 0.36*** |
| **Coalition patterns** |  |  |  |
| 13) % of fights involving coalitions^4^ | 8 | 7 | 8 |
| 14) Conservative coalitions % | 63% | 29% | 85% |
| 15) Bridging coalitions % | 25% | 26% | 13% |
| 16) Revolutionary coalitions % | 12% | 45% | 2% |
| Jonckheere-Terpstra test (C>B>R) | JT=5*** | JT=222 NS | JT=0*** |
| **Triadic awareness patterns** |  |  |  |
| 17) Recipient < Target < Supporter | 63% | 24% | 87% |
| 18) Support given to ‘friend’ | 61% | **54%NS** | 82% |
| **TauKr Correlations** |  |  |  |
| 19) Support Reciprocation (TauKr) | 0.37** | 0.23** | 0.37*** |
| 20) Grooming for Support Received (TauKr) | 0.43** | 0.25** | 0.30*** |
| 21) Support for Grooming Received (TauKr) | 0.21* | 0.31** | 0.23*** |
| 22) Opposition given and opposition received | -0.31* | 0.25*** | -0.42*** |

Patterns among females in GrooFiWorld for several group sizes, sex ratios and degrees of risk aversion. Results represent the average over 10 runs; P-value based on the Bonferroni correction: *p=<0.05; **p=<0.01, ***p=<0.001. In **bold:** results that differ from the full model (in Table 3). ^1^At low intensity of aggression a risk aversion base of 5 implies an actual number of decisions to avoid risks of ~1 (see table 2). ^2^Among all individuals. ^3^1 correlation (5% of 24 correlations) is considered to be a type I error. ^4^For a social facilitation of 10%, the percentage of fights involving coalitions is ~4% and all coalition patterns among females are retained (results not shown, data available on request).
