# Supplementary material for: An Individual-Oriented Model on the Emergence of Support in Fights, Its Reciprocation and Exchange
Source: PLoS One. 2012 May 30;7(5):e37271. doi: 10.1371/journal.pone.0037271 (PMC3364247; doi:10.1371/journal.pone.0037271)
Supplement: Text S1 — Description of the details of the model and of extra results that have been mentioned and discussed in the main text. (DOC) [file pone.0037271.s007.doc]

“An individual-oriented model on the emergence of support in fights, its reciprocation and exchange ”

^1,2^Charlotte K. Hemelrijk and ^1^Ivan Puga-Gonzalez

^1^Behavioural Ecology and Self-organization, Centre for Ecological and Evolutionary Studies, University of Groningen. P.O. Box 11103 9700 CC Groningen, The Netherlands.

^2^To whom correspondence should be addressed. E-mail: [C.K.Hemelrijk@rug.nl](mailto:C.K.Hemelrijk@rug.nl).

**Introduction**

Here we present the details of the model and extra results that have been mentioned and discussed in the main text.

**Results**

Extra results concern the following: A sensitivity analysis of the complete model regarding its behavioural rules, such as the order and assumptions regarding anxiety and risk aversion (Table S1), associations at a group level among grooming, support, opposition and rank (Table S2), correlations for reciprocation and exchange of support partialling out rank and proximity (Table S3), coalition patterns after controlling for immediate reciprocity (Table S4), and sensitivity analysis of parameters of the complete model (group size, sex ratio and degree of risk aversion) (Table S5) .

**Methods**

The model

We use an individual-based model previously developed called GrooFiWorld [1]. It is an extension of a previous model called Dom-World in which individuals group and compete [2-4]. The extension consists of grooming behaviour and that is why we call it ‘GrooFiWorld’ (i.e. Grooming and Fighting).

The space of the ‘world’ is continuous, i.e. individuals are free to move in any direction. They have a certain angle of vision (*VisionAngle*) and a maximum distance of perception (*MaxView*). Individuals are provided with three behavioural tendencies: 1) to group, 2) to perform dominance interactions and 3) to display affiliative behaviour. Why they group or perform dominance interactions is not specified and irrelevant to the model. Individuals groom to reduce anxiety, as suggested for real primates [5-13].

At the start of each run, the individuals occupy random locations within a predefined circumference, *InitRadius*, which is the product of a number times the number of individuals. Their activities are regulated by a timing regime in which each individual receives a random waiting time from a uniform distribution and the individual with the shortest waiting time is activated first. This regime is combined with a biologically plausible timing regime reflecting a kind of ‘social facilitation’ (e.g. see [14]) in which the waiting time of an individual is shortened when a dominance interaction occurs close by (radius of social facilitation, table 2).

Behavioural rules (Figure S1)

Grouping rules.

Whenever an individual does not see another one close by (within its personal space, *PersSpace*), grouping rules come into effect. The individual starts looking for others at greater and greater distances (*NearView* and *MaxView*). If, even then, no one else is in sight, it turns over a *SearchAngle* in order to find others. In this way individuals tend to remain in a group. If, however, an individual spots another one close by, within its personal space (*PersSpace*), a social interaction may take place.

Interactions.

Upon meeting someone else in its personal space (*PersSpace*), an individual first considers whether or not to perform a dominance interaction. This decision depends on the risks involved, whereby risk concerns the chance of losing a fight (i.e. 'Risk-aversion strategy': [3]). A fight is only initiated when the individual expects to win; if defeat is expected, it considers grooming the other. This decision depends on its degree of anxiety: the more anxious an individual is the more incline it is to groom.

Dominance rules.

Dominance interactions are modelled as before [2] and are an extension of the DoDom rules of Hogeweg [15]. A dominance interaction will only take place if an individual expects to be victorious, i.e. it is sensitive to risks. The higher the intensity of aggression of its opponent, the greater its aversion to risk, *RiskAvers*, the more frequently it will perform a ‘mental battle’ before starting an actual fight (Table 2, Equation 1 see parameters). During a ‘mental battle’ individual *i* estimates whether it will win over individual *j*. It is more likely to do so, the higher its rank relative to that of the opponent. This is implemented such that individual *i* compares its dominance value (*Dom_i_*) relatively to that of his opponent *j* (*Dom_j_*) to a random value drawn from a uniform distribution between zero and one (Equation 2).

$w_{i}=\left[\begin{matrix} 1 \frac{DOM_{i}}{DOM_{i}+DOM_{j}}>RND(0,1) \\ 0 else \end{matrix}\right.$ (2)

If its relative dominance value is greater than the random value, it expects to be victorious and will start an actual dominance interaction. During an actual dominance interaction, its outcome is decided by a second comparison of its relative dominance value to a random value.

To reflect the self-reinforcing effects of victory and defeat [16,17], dominance values are updated by increasing the dominance value of the winner and decreasing that of the loser by the same amount (Equation 3). This positive feedback is ‘damped’ because a victory from a higher ranking opponent increases its relative Dom-value only slightly, whereas an (unexpected) success from a lower ranking individual increases its dominance value by a greater amount. This ‘damped’ feedback, thus, allows for dominance reversals. To keep Dom-values positive, their minimum value is, arbitrarily, set at 0.01.

$\begin{matrix} DOM_{i}=DOM_{i}+\left(w_{i}-\frac{DOM_{i}}{DOM_{i}+DOM_{j}}\right)*STEPDOM \\ DOM_{j}=DOM_{j}-\left(w_{i}-\frac{DOM_{j}}{DOM_{i}+DOM_{j}}\right)*STEPDOM \end{matrix}$ (3)

The change in Dom-values is multiplied by a scaling factor, *StepDom*, which represents the intensity of aggression [2,18] (see Parameters and Experimental setup). High values indicate fierce aggression, such as biting, and cause a greater change in Dom-value after a fight. Low *StepDom*-values represent mild aggression, such as threats or slaps and have a lower impact per interaction.

After winning an interaction the winner chases its opponent over a predefined *ChaseDistance* whereby the loser flees over a predefined *FleeingDistance*. Both individuals subsequently turn randomly 45 degrees to right or left.

Grooming behaviour.

Our model is informed by empirical studies which indicate that active and passive grooming serve as an anxiety-reduction mechanism [8,19]. For instance, grooming decreases the heart rate and the rate of self-directed behaviour (i.e. scratching, yawning and shaking), and increases the level of endorphins in the brain reducing, thus, the motivation to groom; and not being groomed for some length of time reduces the concentration of endorphins and increases the motivation to be groomed [5-7,9,11,12,20-22].

In the model, therefore, grooming is induced by the level of *Anxiety* of the individual which ranges from very relaxed to very tense, i.e. from 0 to 1. When an individual decides not to attack other, the individual grooms if its level of *Anxiety* is higher than a random number; otherwise, it displays ‘non-aggressive’ proximity (fig S1). Grooming reduces *Anxiety* (and thus the tendency to groom) in both, the groomee and the groomer. This reduction is stronger in the groomee (*AnxDcrGree*) than in the groomer (*AnxDcrGrmr*). After grooming both partners turn over a small angle (45◦) randomly to the right or left in order to avoid repeated interactions with the same partner. During periods without grooming *Anxiety* increases. This increase is updated after every activation with *AnxInc*. Furthermore, inspired by the observed increase in scratching after a fight in real primates [23], in the model, after a fight *Anxiety* increases with *AnxIncFght* in both opponents. At the beginning of the simulation all individuals start with the same *Anxiety* value, i.e. 0.5, *InitAnx*.

Sensitivity analysis of the behavioural rules of the individuals:

We studied the consequences of the following manipulations of behavioural rules: a) aggression and grooming in the reversed order of that of the complete model. In this case, individuals considered first whether or not to groom the other and subsequently whether or not to attack, b) considering grooming and aggression in a random order. Here individuals had a random chance of 50% of first considering to groom or to attack, c) actively always grooming others upon estimating to lose a fight, thus, individuals did not take into account their anxiety level like they did in the complete model, d) considering attacking another by chance (namely 15%, the same percentage as in the complete model), thus, not depending on the chance to win as individuals did in the complete model.

References

1. Puga-Gonzalez I, Hildenbrandt H, Hemelrijk CK (2009) Emergent patterns of social affiliation in primates, a model. Plos Computational Biology 5: e1000630. doi:10.1371/journal.pcbi.1000630.

2. Hemelrijk CK (1999) An individual-oriented model on the emergence of despotic and egalitarian societies. Proceedings of the Royal Society London B: Biological Sciences. 266: 361-369.

3. Hemelrijk CK (2000) Towards the integration of social dominance and spatial structure. Animal Behaviour 59: 1035-1048.

4. Hemelrijk CK (2005) A process-oriented approach to the social behaviour of primates. In: Hemelrijk CK, editor. Self-organisation and evolution of social systems. Cambridge, UK: Cambridge University Press. pp. 81-107.

5. Meller RE, Keverne EB, Herbert J (1980) Behavioral and endocrine effects of naltrexone in male talapoin monkeys. Pharmacology Biochemistry and Behavior 13(5): 663-672.

6. Fabre-Nys C, Meller RE, Keverne EB (1982) Opiate antagonists stimulate affiliative behavior in monkeys. Pharmacology Biochemistry and Behavior 16(4): 653-659.

7. Boccia ML (1987) The physiology of grooming - A test of the tension reduction hypothesis. Am J Primatol 12(3): 330-330.

8. Schino G, Scucchi S, Maestripieri D, Turillazzi PG (1988) Allogrooming as a tension-reduction mechanism: A behavioral approach. American Journal of Primatology 16: 43-50.

9. Keverne EB, Martensz ND, Tuite B (1989) Beta-endorphin concentrations in cerebrospinal-fluid of monkeys are influenced by grooming relationships. Psychoneuroendocrinology 14(1-2): 155-161.

10. Schino G, Troisi A (1992) Opiate receptor blockade in juvenile macaques: Effect on affiliative interactions with their mothers and group companions. Brain Research 576(1): 125-130.

11. Martel FL, Nevison CM, Simpson MJA, Keverne EB (1995) Effects of opioid receptor blockade on the social behavior of rhesus monkeys living in large family groups. Developmental Psychobiology 28(2): 71-84.

12. Graves FC, Wallen K, Maestripieri D (2002) Opioids and attachment in rhesus macaque (*macaca mulatta*) abusive mothers. Behav Neurosci 116(3): 489-493.

13. Sannen A, van Elsacker L, Eens M (2004) Effect of spatial crowding on aggressive behaviour in a bonobo colony. Zoo Biology 23: 383-395.

14. Galef BG, Jr (1988) Imitation in animals: History, definitions, and interpretation of data from the psychological laboratory. In: Zentall T, Galef B, editors. Social learning: Psychobiological and biological perspectives. Hillsdale, New Jersey: Erlbaum. pp. 3-28.

15. Hogeweg P (1988) MIRROR beyond MIRROR, puddles of LIFE. In: Langton C, editor. Artificial life, SFI studies in the sciences of complexity. Redwood City, California: Adisson-Wesley Publishing Company. pp. 297-316.

16. Hsu Y, Wolf LL (1999) The winner and loser effect: Integrating multiple experiences. Animal Behaviour 57: 903-910.

17. Hemelrijk CK, Wantia J, Isler K (2008) Female dominance over males in primates: Self-organisation and sexual dimorphism. PLoS ONE 3(7): e2678.

18. Hemelrijk CK (1998) Risk sensitive and ambiguity reducing dominance interactions in a virtual laboratory. : 255-262.

19. Shutt K, MacLarnon A, Heistermann M, Semple S (2007) Grooming in barbary macaques: Better to give than to receive? Biol Lett 3(3): 231-233.

20. Boccia ML, Reite M, Laudenslager M (1989) On the physiology of grooming in a pigtail macaque. Physiology & Behavior 45: 667-670.

21. Aureli F, van Schaik CP (1991) Postconflict behaviour in long-tailed macaques (*macaca fascicularis*): II. coping with the uncertainty. Ethology 89: 101-114.

22. Das M, Penke Z, van Hooff JARAM (1998) Postconflict affiliation and stress-related behaviour of long-tailed macaque aggressors. International Journal of Primatology 19(1): 53-71.

23. Aureli F, Cords M, Van Schaik CP (2002) Conflict resolution following aggression in gregarious animals: A predictive framework. Animal Behaviour 64: 325-343.
